# The rate of short-term revisits after diagnosis of non-specific abdominal pain is similar for surgeons and emergency physicians - results from a single tertiary hospital emergency department

**DOI:** 10.1186/s13049-020-00751-8

**Published:** 2020-07-01

**Authors:** Leena Saaristo, Mika T. Ukkonen, Johanna M. Laukkarinen, Satu-Liisa K. Pauniaho

**Affiliations:** 1 Department of Surgery, Seinäjoki Central Hospital, Seinäjoki, Finland; 2 Faculty of Medicine and Life Sciences, University of Tampere, Tampere, Finland; 3 Department of Gastroenterology and Alimentary Tract Surgery, Tampere University Hospital, Finland. Faculty of medicine and health technology, Tampere University, Tampere, Finland; 4 Emergency Division, Tampere University Hospital, Tampere, Finland; 5 Department of Adolescent Psychiatry, Tampere University Hospital, Tampere, Finland

## Abstract

**Background:**

Acute abdominal pain can be a diagnostic challenge even for experienced surgeons. Delayed diagnosis can lead to higher morbidity, mortality and increased costs. While readmission rate has been used to evaluate quality of surgical care, studies addressing the issue in emergency departments (ED) are rare. The role of emergency physicians in the care of patients with abdominal pain is increasing in many European countries, including Finland. It is not known whether this has an effect on the number of readmissions.

Here we evaluate whether the increasing role of emergency physicians in examining patients presenting with abdominal pain has affected the rate of short-term revisits among patients with non-specific abdominal pain (NSAP).

**Methods:**

We identified consecutive ED patients receiving a diagnosis of NSAP 1.1. 2015–31.12.2016 in the ED of Tampere University Hospital. Those revisiting the ED within 48 h were selected for further analysis. Data were obtained from electronic medical records. We compared the outcomes of those initially examined by surgeons and by emergency physicians.

**Results:**

During the study period, 173,630 patients visited our ED, of whom 6.1% (*n* = 10,609) were discharged with a diagnosis of NSAP. Only 3.0% of patients revisited the ED, 0.7% required hospitalization and 0.06% immediate surgery. The short-term revisit rates among those originally examined by surgeons and by emergency physicians were similar, 2.8 and 3.2% respectively (*p* = 0.193).

**Conclusions:**

The rate of short-term revisits in patients with NSAP was altogether low. The increasing role of emergency physicians in the care of acute abdominal patients did not affect the revisit rate.

## Background

Acute abdominal pain is among the most common reasons for emergency department (ED) visits [1–3], and can be a diagnostic challenge even for experienced surgeons [4]. In most cases the cause of the abdominal pain remains unclear and the patients are often discharged with a diagnosis of non-specific abdominal pain (NSAP) [3, 5, 6]. Some of these patients return to the ED due to persistence or acerbation of the symptoms, and some are later diagnosed with conditions requiring emergency surgery. Delay in the diagnostics or misdiagnoses may be associated with a higher risk of more complicated acute conditions and subsequently with a higher risk of adverse events, longer hospitalization and higher costs of care [7–10]. Revisits also cause a burden on already overcrowded EDs. Consequently, methods to prevent unnecessary revisits are needed.

While readmission rate is among the parameters commonly used to evaluate the quality of surgical care [11–13], studies in emergency medicine concerning revisits are rare [3, 14–16]. In this study we focused on short-term revisits among patients receiving a diagnosis of NSAP. Moreover, we wanted to evaluate whether the shift towards these patients being seen by emergency physicians rather than surgeons has had an impact in the revisit rates or possible complications.

The primary aim was to ascertain the rate of unplanned ED revisits after NSAP diagnosis. The secondary aim was to ascertain whether the increasing role of emergency physicians examining patients with acute abdominal pain has resulted in a higher rate of short-term revisits.

## Methods

In this retrospective cohort study over a period of 2 years (January 2015 to December 2016) medical records of each consecutive patient with NSAP attending Tampere University Hospital ED, a high-volume, collaborative ED were reviewed. Patients were identified by retrieving all cases associated with the International Statistical Classification of Diseases and Related Health Problems (10th revision, ICD-10) with a diagnosis of any subclass of “R10”. Of these, patients returning to the ED within the following 48 h were included for further analysis.

We obtained the data by reviewing each case, including laboratory and imaging results. Demographic information and relevant medical history were recorded for each patient.

Tampere University Hospital is a tertiary care unit with a catchment area of approximately 500,000 inhabitants. All emergency cases within the city are admitted to the study hospital. Referrals to ED from general practitioner are not required. During the study period, the ED went through an organizational change. In 2015 emergency patients with a referral were examined either by a surgeon-in-training or by a specialist in surgery. Patients without a referral were seen by a primary health care physician or emergency physician, with the option to refer the patient for surgical assessment. In 2016, the organization was changed and emergency physicians, including specializing ED doctors, specialists in emergency medicine and emergency-work oriented general practitioners started working side by side with the surgeons, examining patients with or without a referral. This gave us an opportunity to study revisits after NSAP diagnosis set by a surgeon or by an emergency physician during the study period. The follow-up data was available for all patients.

This study is done according to the STROBE guidelines (www.strobe-statement.org).

### Statistical analysis

Statistical analysis was performed using SPSS for Windows statistical software version 22 with Chi square test to compare categorical data, Mann–Whitney U-test and Kruskal–Wallis H-test to compare the medians between groups in nonparametric variables. Statistical significance was set at *p* < 0.05.

### Ethical aspects

The study was performed according to the Helsinki Declaration and institutional review board approval was duly obtained (R16006)**.**

## Results

**Fig. 1:**
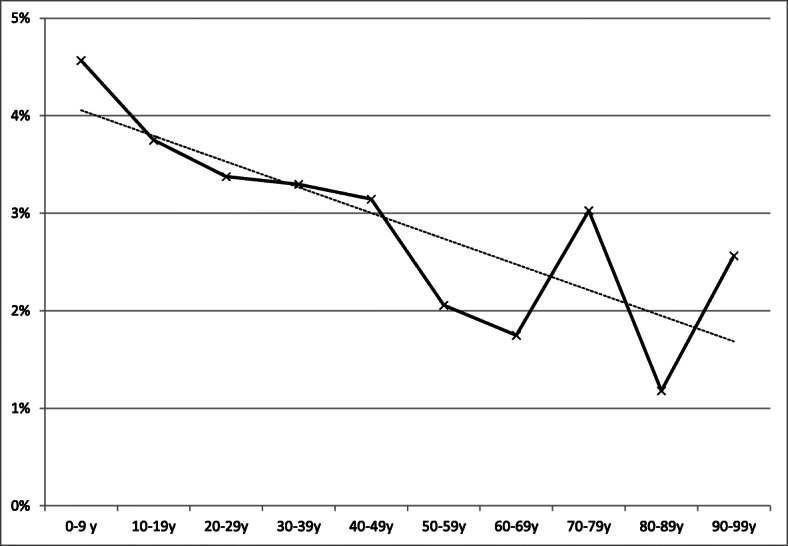
Rate of short-term emergency department revisits among those with a diagnosis of non-specific abdominal pain in different age groups. Tampere University Hospital, 2015–2016

During the two-year study period, a total of 173,630 patients visited the ED, of whom 6.1% (*n* = 10,609; median age 38 years, 0–100 years; 60% female) were discharged with a diagnosis of NSAP. Of these patients 3.0% (*n* = 313; median age 32 years, 0–98 years; 64% female) revisited the ED within the following 48 h, with no significant change observed in the revisiting rate during the study (2.8% in 2015 vs. 3.1% in 2016, *p* = 0.798). The majority (94%) of patients returned due to persisting symptoms, while the rest presented with new symptoms. Younger age was associated with increased likelihood of revisits, as illustrated in Fig. 1. Two thirds (67%) of NSAP patients did not receive a specific diagnosis on the second visit, while typical specific new diagnoses included acute cholecystitis (6.1%), appendicitis (3.8%) and pancreatitis (3.6%). Those diagnosed with a gynaecological condition, e.g. ruptured ovarian cyst, amounted to 5.4%. Demographic characteristics and distribution of diagnoses in the study population are shown in Table 1.

**Table 1:** Demographic characteristics of NSAP patients readmitted to ED within 48 h of the index visit

	Year 2015 *n* = 85,149	Year 2016 *n* = 88,481	All patients *n* = 173,630
NSAP patients	6024 (7.1%)	4585 (5.2%)	10,609 (6.1%)
Readmitted within 48 h	170 (2.8%)	143 (3.1%)	313 (3.0%)
Age, median (range)	36 y (0–98 y)	28 y (4–95 y)	32 y (0–98 y)
Gender, female, n (%)	105 (62%)	94 (66%)	199 (54%)
Comorbidities, n (%)	72 (42%)	70 (49%)	142 (45%)
Cardiovascular	33 (19%)	24 (17%)	57 (18%)
Pulmonary	17 (10%)	10 (7.0%)	27 (8.6%)
Psychiatric	15 (8.8%)	13 (9.1%)	28 (8.9%)
Diabetes	8 (4.7%)	14 (9.8%)	22 (7.0%)
Neurologic	3 (1.8%)	12 (8.4%)	15 (4.7%)
Alcoholism	6 (3.5%)	4 (2.8%)	10 (3.2%)
Dementia	3 (1.8%	1 (0.7%)	4 (1.2%)
Medication, n (%)	92 (54%)	59 (41%)	151 (48%)
Anticoagulation	11 (6.5%)	13 (9.1%)	24 (7.7%)
Corticosteroids	3 (1.8%)	1 (0.7%)	4 (1.2%)
Previous abd. Surgery	46 (27%)	30 (21%)	76 (24%)
Frequent users (≥5 admissions/year)	20 (12%)	17 (12%)	37 (12%)
Specific diagnosis	60 (35%)	44 (31%)	104 (33%)
Acute cholecystitis	16 (9.4%)	3 (2.1%)	19 (6.1%)
Gynaecological	11 (6.5%)	6 (4.2%)	17 (5.4%)
Acute appendicitis	6 (3.5%)	6 (4.2%)	12 (3.8%)
Acute pancreatitis	4 (2.4%)	6 (4.2%)	10 (3.2%)
Urinary tract stone disease	3 (1.8%)	0 (0.0%)	3 (1.0%)
Diverticulitis	1 (0.6%)	2 (1.4%)	3 (1.0%)
Abdominal emergencies ^a^	3 (1.8%)	4 (2.8%)	7 (2.2%)
Miscellaneous	16 (9.4%)	17 (12%)	33 (11%)
Non-specific diagnoses ^b^	110 (65%)	99 (69%)	209 (67%)

^a^ Abdominal emergencies requiring immediate treatment (e.g. acute mesenteric ischaemia, bowel perforation)

^b^ Other conditions, e.g. gastroenteritis, extra-abdominal conditions (e.q. pneumonia)

**Fig. 2:**
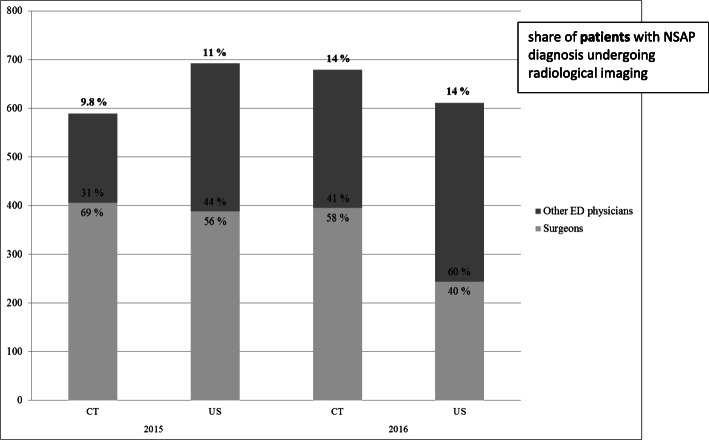
Number of CT and ultrasound examinations requested by surgeons and emergency physicians. Upper percentage shows the share of patients with a diagnosis of non-specific abdominal pain undergoing radiological imaging during the emergency department visit

Of the patients revisiting the ED, 67% were discharged with NSAP diagnosis, 16% were admitted to the hospital for surgical and 9.3% for conservative treatment. Fifty-four percent of those requiring surgical and 29% of those requiring conservative treatment on in-patient wards required radiological studies to reach a diagnosis. Only a minority of these patients had undergone radiological examinations at the first ED presentation, as illustrated in Fig. 2. Both WBC and CRP values during the index and second admission were higher among hospitalized patients than among those discharged home. There was a wide variation in the laboratory findings; i.e. the values were within normal limits in some patients who required surgery and greatly elevated in some patients with an NSAP diagnosis. Comparisons of laboratory findings and the use of radiological imaging in different patient groups are shown in Table 2.

**Table 2:** Laboratory findings and utilization of radiological imaging on the first and second visit in patients with NSAP

	Discharged with NSAP (*n* = 208)	Admitted to the hospital for	
		Conservative treatment (*n* = 29)	Surgical treatment (*n* = 50)
Age, median (min-max)	28 y (0–98 y)	49 y (23–95 y)	46 y (10–85 y)
1st visit			
WBC (10^9^/l), median (min-max) ^2^	8.1 (3.0–23)	10 (5.8–17)	10 (3.0–18)
CRP (mg/l), median (min-max)	1.9 (1.0–96)	4.5 (1.0–163)	3.0 (1–144)
Ultrasonography, n (%)	6 (2.9%)	3 (10%)	3 (6.0%)
CT scan, n (%)	2 (1.0%)	2 (6.9%)	0 (0.0%)
2nd visit			
WBC (10^9^/l), median (min-max) ^2^	7.6 (3.2–18)	9.7 (3.5–21)	10.1 (3.2–25.9)
CRP (mg/l), median (min-max) ^2^	2.3 (1–234)	32 (1.0–329)	63.8 (1–414)
Ultrasonography, n (%)	42 (20%)	6 (21%)	20 (40%)
CT scan, n (%)	26 (13%)	11 (38%)	18 (36%)
Radiological imaging required	–	13 (29%)	27 (54%)

Statistically significant difference: ^1^*p*-value 0.05–0.001, ^2^ p-value < 0.001

*CRP C* reactive protein, *WBC* white blood cells, *CT* computed tomography, *NSAP* non-specific abdominal pain

An acute condition requiring immediate surgical treatment (i.e. vascular emergencies or bowel perforation) was diagnosed on 0.07% of the patients (*n* = 7). Six out of seven of those patients with surgical emergencies were examined by emergency physicians during the index admission. While 71% of these patients (*n* = 5) would have required radiological imaging to reach a diagnosis, none had undergone these examinations during the index visit.

A total of 58% of those with NSAP diagnosis (*n* = 6153) underwent examination by emergency physicians and the rest (*n* = 4455) by surgeons or surgical registrars. After implementing the new collaborative ED system and the emergency medicine residency programme, the share of NSAP patients examined by surgeons diminished from 49 to 33% corresponding to an increasing role of emergency physicians dealing with seeing patients with acute abdominal pain. The rates of short-term revisits among those examined by surgeons and by emergency physicians were 2.8% (2.4% in 2015 and 3.1% in 2016) and 3.2% (3.5, 2.6%) respectively (*p* = 0.193).

## Discussion

In this study, we focused on short-term revisits by patients receiving a diagnosis of NSAP in an attempt to assess whether the shift towards these patients being seen by emergency physicians rather than by surgeons affected the revisit rates or possible complications. We found that revisits due to NSAP were in general rare and that the rate did not increase after emergency physicians started to examine the majority of these patients in the ED.

In this study we show that the diagnostic accuracy in acute abdominal pain in our unit was overall good with a limited number of short-term revisits associated with an NSAP diagnosis. Only 3% of NSAP patients revisited the ED within the following 48 h and less than 1% received a diagnosis requiring admission either for surgical or for conservative treatment. Only six out of 10,000 NSAP patients suffered from conditions requiring immediate attention. The increasing role of emergency physicians in examining patients with acute abdominal pain in 2016 was not associated with a higher rate of short-term revisits. However, almost all of the patients discharged during the first visit who later required immediate surgery, were examined by emergency physicians. Consequently the rate of missed emergencies could have been reduced if these patients had been examined by surgeons during the first admissions. Morbidity and mortality meetings should be systematically used in case of missed severe diagnoses for educational and quality improvement purposes.

Our results concur with those of earlier studies, where the return visit rate has ranged from 1.9 to 3.5% [17, 18]. However, these studies have not focused on patients with acute abdominal pain but on all ED patients. Furthermore, revisits have been considered more common among patients with abdominal symptoms [3]. Consequently, compared to earlier studies, we consider our rate of revisits (3.0%) to be surprisingly low.

Almost all the returning patients had persisting symptoms, and the rest presented with onset of new symptoms. More than two-thirds of these patients received the same non-specific diagnosis after the second visit and if all NSAP patients are included, only 1% received a new diagnosis. The most common specific diagnoses were acute appendicitis, acute pancreatitis and acute cholecystitis. However, it is not uncommon for the first symptoms during the onset of an acute condition to be non-specific, i.e. visceral pain and nausea during the first hours after the onset of the condition. In these subacute conditions, a short diagnostic delay is rarely associated with poorer outcomes [7, 19].

Radiological imaging was rarely used during the index visit and use of imaging among NSAP patients was not associated with lower likelihood of further visits. Using radiological imaging on all NSAP patients would increase the costs and exposure to radiation and could potentially lead to false-negative imaging due to early presentation. While some patients were misdiagnosed during the index visit and subsequently required surgical treatment, the delay to surgery seldom affected the outcome. Consequently, we consider our results acceptable. However, we emphasize that training emergency physicians to deal with patients with acute abdominal pain, and to spot “red flags” in patients with surgical emergencies is crucial. While emergency physicians have an increasing role in evaluating patients with acute abdominal pain, access to surgeons in a consultative capacity for these patients is essential now, as it will be in the future.

There were some obvious limitations to our study. The study was retrospective and thus the amount of information obtained from the medical records was limited. Laboratory results and findings in radiological imaging were available for all patients, but the case notes were not always structured. The strength of the study, however, was that our hospital ED covers all levels of ED care throughout the entire hospital district. Thus, the patients will return to our ED for revisits. The follow-up data was available for all patients. Furthermore, to the best of our knowledge, this is the first study to focus on the revisit rate among ED patients with NSAP. Finally, we have shown that regardless of the establishment of the emergency medicine residency programme, the number of unnecessary revisits did not increase.

## Conclusion

Unplanned revisits may cause a burden on already overcrowded EDs. However, we found that after the establishment of the collaborative emergency department and emergency medicine residency programme diagnostic accuracy among NSAP patients, often considered difficult to diagnose, was overall good. The increasing trend for emergency physicians to examine more and more patients with abdominal pain has not increased the revisit rates of NSAP patients. We emphasize the absolute need for educating emergency physicians in diagnosing surgical emergencies.

## Data Availability

Not applicable.

## Abbreviations

NSAP: No specific abdominal pain
ED: Emergency department
WBC: White blood cell count
CRP: C reactive protein

## References

[1] Bruns BR, Tesoriero R, Narayan M, Klyushnenkova EN, Chen H, Scalea TM (2015). Emergency general surgery: defining burden of disease in the state of Maryland. Am Surg.

[2] Kamin RA, Nowicki TA, Courtney DS, Powers RD (2003). Pearls and pitfalls in the emergency department evaluation of abdominal pain. Emerg Med Clin North Am.

[3] Cervellin G, Mora R, Ticinesi A, Meschi T, Comelli I, Catena F (2016). Epidemiology and outcomes of acute abdominal pain in a large urban emergency department: retrospective analysis of 5,340 cases. Ann Transl Med.

[4] Kiewiet J, Gans S, Luitse J, van Westreenen H, Lamme B, Welling L (2016). Diagnostic accuracy of surgeons and trainees in assessment of patients with acute abdominal pain. Br J Surg.

[5] Fagerstrom A, Paajanen P, Saarelainen H, Ahonen-Siirtola M, Ukkonen M, Miettinen P (2017). Non-specific abdominal pain remains as the most common reason for acute abdomen: 26-year retrospective audit in one emergency unit. Scand J Gastroenterol.

[6] Eskelinen M, Lipponen P (2012). Usefulness of history-taking in non-specific abdominal pain: a prospective study of 1333 patients with acute abdominal pain in Finland. In Vivo.

[7] Teixeira PG, Sivrikoz E, Inaba K, Talving P, Lam L, Demetriades D (2012). Appendectomy timing: waiting until the next morning increases the risk of surgical site infections. Ann Surg.

[8] Davies M, Davies C, Morris-Stiff G, Shute K (2007). Emergency presentation of abdominal hernias: outcome and reasons for delay in treatment - a prospective study. Ann R Coll Surg Engl.

[9] Kachalia AJD, Gandhi TK, Puopolo ALBSN, Yoon C, Thomas EJ, Griffey R (2007). Missed and delayed diagnoses in the emergency department: a study of closed malpractice claims from 4 liability insurers. Ann Emerg Med.

[10] Cappendijk VC, Hazebroek FWJ (2000). The impact of diagnostic delay on the course of acute appendicitis. Arch Dis Child.

[11] Havens JM, Olufajo OA, Cooper ZR, Haider AH, Shah AA, Salim A (2016). Defining rates and risk factors for readmissions following emergency general surgery. JAMA Surgery.

[12] PKa G, FernandesTaylor S, BBS R, TLa E, CKa K (2014). Unplanned readmissions after vascular surgery. J Vasc Surg.

[13] Bliss LA, Maguire LH, Chau Z, Yang CJ, Nagle DA, Chan AT (2015). Readmission after resections of the Colon and Rectum: predictors of a costly and common outcome. Dis Colon Rectum.

[14] Patterson BW, Venkatesh AKMHS, AlKhawam L, Pang PS, Carpenter CR (2015). Abdominal computed tomography utilization and 30-day Revisitation in emergency department patients presenting w?With abdominal pain. Acad Emerg Med.

[15] Sabbatini AK, Kocher KE, Basu A, Hsia RY (2016). In-hospital outcomes and costs among patients hospitalized during a return visit to the emergency department. JAMA.

[16] Wu C, Wang F, Chiang Y, Chiu Y, Lin T, Fu L (2010). Unplanned emergency department revisits within 72 hours to a secondary teaching referral hospital in Taiwan. J Emerg Med.

[17] Verelst S, Pierloot S, Desruelles D, Gillet J, Bergs J (2014). Short-term unscheduled return visits of adult patients to the emergency department. J Emerg Med.

[18] Cheng S, Wang H, Lee C, Tsai T, Hung C, Wu K (2013). The characteristics and prognostic predictors of unplanned hospital admission within 72 hours after ED discharge. Am J Emerg Med.

[19] Mayumi T, Okamoto K, Takada T, Strasberg SM, Solomkin JS, Schlossberg D, et al. Management bundles for acute cholangitis and cholecystitis. J Hepato-biliary-Pancreatic Sci. 2018;25(1):96–100. 10.1002/jhbp.519 29090868

